# Mining Sudanese Medicinal Plants for Antiprotozoal Agents

**DOI:** 10.3389/fphar.2020.00865

**Published:** 2020-06-09

**Authors:** Abdelhalim Babiker Mahmoud, Pascal Mäser, Marcel Kaiser, Matthias Hamburger, Sami Khalid

**Affiliations:** ^1^Parasite Chemotherapy Unit, Swiss Tropical and Public Health Institute, Basel, Switzerland; ^2^Faculty of Science, University of Basel, Basel, Switzerland; ^3^Faculty of Pharmacy, University of Khartoum, Khartoum, Sudan; ^4^Faculty of Pharmacy, University of Science and Technology, Omdurman, Sudan

**Keywords:** HPLC activity profiling, drug discovery, Sudan, medicinal plant, Trypanosoma, Leishmania, Plasmodium

## Abstract

Neglected tropical diseases are major health hazards in developing countries. Annually, up to 30 million people are affected by either Chagas disease, African trypansomiasis or leishmaniasis, and more than 200 million by malaria. Most of the currently available drugs have drawbacks in terms of toxicity, limited oral availability, development of resistance, or non-affordability. Tropical plants of the arid zones are a treasure chest for the discovery of bioactive secondary metabolites. This study aims to compile Sudanese medicinal plants, validate their antiprotozoal activities, and identify active molecules. We have performed a survey of medicinal plants of Sudan and selected 62 that are being used in Sudanese traditional medicine. From these, we collected materials such as leaves, stem, bark, or fruit. The plant materials were extracted in 70% ethanol and further fractionated by liquid-liquid partitioning using solvents of increasing polarity. This resulted in a library of 235 fractions. The library was tested *in vitro* against *Plasmodium falciparum* (erythrocytic stages), *Trypanosoma brucei rhodesiense* (bloodstream forms), *Trypanosoma cruzi* (intracellular amastigotes), and *Leishmania donovani* (axenic amastigotes). Active fractions were also tested for cytotoxicity. Of the 235 fractions, 125 showed growth inhibitory activity >80% at 10 μg/ml, and >50% at 2 μg/ml against at least one of the protozoan parasites. *Plasmodium falciparum* was the most sensitive of the parasites, followed by *T. b. rhodesiense* and *L. donovani*. Only few hits were identified for *T. cruzi*, and these were not selective. Contrary to expectation based on phylogeny, but in agreement with previous results, a large number of extracts displayed mutual activity against *T. brucei* and *P. falciparum*. HPLC-based activity profiling for selected active extracts was performed to identify the bioactive principles. Active compounds identified by dereplication were guieranone A from *Guiera senegalensis* J.F.Gmel.; pseudosemiglabrin from *Tephrosia apollinea* (Delile) DC; ellagic acid and quercetin from *Terminalia leiocarpa* (DC.) Baill.; and catechin, ethyl gallate, and epicatechin gallate from *Vachellia nilotica* (L.) P.J.H.Hurter & Mabb. Also the extracts of *Croton gratissimus* var. *gratissimus* and *Cuscuta hyalina* Roth ex Schult. exhibited promising antitrypanosomatid activity. This assessment provides a comprehensive overview of Sudanese medicinal plants and supports the notion that they are a potential source of bioactive molecules against protozoan parasites.

## Introduction

Infections by protozoan parasites remain to be among the most devastating causes of mortality in the tropics. The trypanosomatids are a large family of flagellated protozoa, some of which cause neglected tropical diseases of high public health relevance and socio-economic impact (WHO, n.d.; Filardy et al., 2018). These are *Trypanosoma cruzi* (Chagas’ disease), *T. brucei gambiense*, and *T. b. rhodesiense* (human African trypanosomiasis or sleeping sickness), and *Leishmania* spp. (different kinds of leishmaniasis) (Stuart et al., 2008). The apicomplexan parasite *Plasmodium falciparum* is the causative agent of malaria tropica, the most dangerous form of malaria, which—despite the successes by various international bodies and philanthropic organizations—still claims an annual death toll of 435,000 (World Health Organisation, 2018). These diseases disproportionally affect the poor and vulnerable populations (WHO Expert Committee on Malaria: Twentieth Report, n.d.), calling for action to improve global well-being. A key element of the fight against protozoan neglected tropical diseases and malaria is the discovery of novel chemotherapeutic agents.

While the incidence of human African trypanosomiasis is at a historic low and a new drug, fexinidazole (Mesu et al., 2018), has recently received positive opinion by the European Medicines Agency, the prospects are slightly gloomy for other protozoal diseases. Chagas’ disease has reached global dimensions (“WHO | Epidemiology” n.d.), and leishmaniasis as well (“Leishmaniasis” n.d.). Sudan has the highest incidence of leishmaniasis in sub-Saharan countries, with 15,000–20,000 new cases per annum (Hotez and Kamath, 2009). The successful treatment of malaria is threatened by artemisinin-resistant mutants of *P. falciparum*, first reported from Southeast Asia (Ariey et al., 2014; Straimer et al., 2015; Ménard et al., 2016) and, more recently, also from Africa (Lu et al., 2017).

Plants are still considered as important sources for the discovery of novel bioactive molecules. Plants secondary metabolism represents a huge and unique reservoir of chemical diversity, which may serve as a source of new drugs, either directly or after optimization by medicinal chemistry. Independent chemoinformatic analyses have consistently shown that natural products often exhibit unique features, a high degree of structural diversity, and drug- or lead-like structural properties (Feher and Schmidt, 2003; Schmidt et al., 2012; Pascolutti et al., 2015).

A retrospective analysis showed that approximately 50% of drugs approved within the last 30 years are derived, directly or indirectly, from natural products, whereby plant derived compounds played an important role (Newman and Cragg, 2016).

Sudan’s biodiversity coupled with a deeply rooted ethno-botanical heritage is an untapped reservoir for the discovery of new bioactive natural products. Here we performed a survey of plants from Sudan that are used in traditional medicine, with a focus on malaria and neglected tropical diseases caused by protozoa. On the basis of this survey a library of plant extracts was assembled and screened against trypanosomatid parasites and *P. falciparum*. Active compounds in the most promising extracts were tracked with the aid of an activity-driven approach.

## Materials and Methods

### Preparation of a Library of Plant Extracts

A total of 62 plants reputed as antiparasitic in traditional medicine in Sudan were solicited from the repository of the Faculty of Pharmacy, University of Science & Technology. The plants belonged to 35 different families, of which the Combretaceae, Leguminosae, Verbenaceae, Lamiaceae, and Compositae were the most frequent. Where available, different parts of a given plant species were included in the study.

The taxonomic identity was confirmed by the Medicinal and Aromatic Plants Research Institute, Sudan. Voucher specimens (USTH 01-USTH 62) have been deposited at the Herbarium of the faculty of Pharmacy, University of Science and Technology, Omdurman, Sudan.

Dried plant material was milled to coarse powder in a hammer mill. 100-500 g of powdered material was extracted for 24 h with 500 ml of 70% ethanol in a magnetic rod stirrer. Extracts were filtered through Whatman no. 1 filter paper and concentrated by solvent removal in a rotary vacuum evaporator. Crude extracts were suspended in water and partitioned consecutively with petroleum ether, chloroform, ethyl acetate, and n-butanol. Crude extracts and their respective fractions were allowed to dry at room temperature, weighed, and reconstituted in DMSO (10 mg/ml) to serve as stock solutions for antiparasitic testing. This resulted in a library of 235 samples.

### HPLC Analyses and Microfractionation

HPLC analyses were performed on a Shimadzu HPLC system equipped with photo diode array detector (PDA) (SPD-M20A, Shimadzu), evaporative light scattering detector (ELSD) (3300, Alltech), and an electrospray ionization mass spectrometer (ESIMS) (LCMS-8030, Shimadzu). LabSolutions software was used for data acquisition and processing. The separation was performed on a C18 SunFire column (3.0 × 150 mm; 3.5 μm; Waters).

Microfractionation of the active samples was carried out by analytical RP-HPLC on an LC-MS 8030 system (Shimadzu) connected with an FC204 fraction collector (Gilson). For each fraction, a solution of 10 mg/ml was prepared in DMSO. A total of three injections were performed: 2 × 35 μl with only UV detection (254 nm) for collection (0.7 mg of fraction in total) and 1 × 35 μl with UV-ELSD-ESIMS detection without collection.

The mobile phase consisted of water with 0.1% formic acid (A) and acetonitrile with 0.1% formic acid (B). The gradient was 5% to 100% B in 30 min, followed by washing with 100% B for 10 min. The flow rate was 0.4 ml/min. Fractions of 1 min each were collected from minute 1 to minute 40, resulting in 40 microfractions in total. Microfractions of two successive injections of a given sample were collected into the corresponding wells of a 96-deepwell plate. Plates were then dried in a Genevac EZ-2 evaporator (Potterat and Hamburger, 2013; Potterat and Hamburger, 2014).

### Activity Testing Against *Trypanosoma brucei rhodesiense*

*In vitro* activity was tested against bloodstream-form *T. b. rhodesiense* STIB 900, which had been obtained in 1982 from a Tanzanian patient and adapted to axenic culture (Baltz et al., 1985). The culture medium was MEM supplemented with 25 mM HEPES, 1 g/L additional glucose, 1% MEM nonessential amino acids, 0.2 mM 2-mercaptoethanol, 1 mM Na-pyruvate, and 15% heat inactivated horse serum. In the two-concentration assay, 50 µl medium containing the corresponding samples concentration (10 µg/ml or 2  µg/ml) was added to the wells of a 96 well plate. For the IC_50_ determination, a 50-µl medium was added to each well, and a serial sample dilution of 11 threefold dilution steps covering a range from 100 to 0.002 μg/ml were prepared. Then 10^4^ *T. b. rhodesiense* in 50 µl medium was added to the wells, and the plate was incubated for 72 h at 37 °C in a humidified atmosphere of 5% CO_2_. A 10-µl resazurin solution (12.5 mg resazurin dissolved in 100 ml distilled water) was added to each well and incubated for a further 2  to 4 h (Räz et al., 1997). Plate reading was performed in a Spectramax Gemini XS microplate fluorometer (Molecular Devices Corporation) using an excitation wavelength of 536 nm and emission wavelength of 588 nm. Melarsoprol was used as reference drug. Final in-test DMSO concentration did not exceed 1%. All assays were performed in two independent replicates at least.

### Activity Testing Against *Leishmania donovani*

*L. donovani* amastigotes strain MHOM/ET/67/L82 were grown in axenic culture in SM medium at pH 5.4 with 10% heat-inactivated fetal bovine serum, at 37 °C in a humidified atmosphere of 5% CO_2_. In the two-concentration assay, 50 µL medium containing the corresponding samples concentration (10 or 2  µg/ml) was added to the wells of a 96 well plate. For the IC_50_ determination, 50 µl medium was added to each well and a serial sample dilution of eleven 3-fold dilution steps covering a range from 100 to 0.002 μg/ml were prepared. Then 10^5^ *L. donovani* amastigotes in 50 µl medium were added to the wells, and the plate was incubated for 72 h at 37 °C in a humidified atmosphere of 5% CO_2_. After 72 h of incubation, 10 μl of resazurin solution were added to each well and the plates incubated for another 2 h (Mikus and Steverding, 2000). Plate reading was performed as described for *T. brucei*. Miltefosine was used as reference drug. Final in-test DMSO concentration did not exceed 1%. All assays were performed in two independent replicates at least.

### Activity Testing Against *Trypanosoma cruzi*

All tests were performed with the *T. cruzi* Tulahuen strain C2C4, which expresses the β-galactosidase (*LacZ*) gene (Buckner et al., 1996). L6 rat skeletal myoblasts served as host cells. Cultures were maintained in RPMI 1640 medium supplemented with 10% FBS and 1.7 µM l-glutamine at 37°C in a humidified atmosphere of 5% CO2. Host cells were seeded in 96-well microtitre plates, 2 × 10^3^ per well in 100-µl medium. After 24 h, 50 µl of a suspension of 1 × 10^5^/ml trypomastigote *T. cruzi* were added. The medium was replaced at day 4, test samples were added, and the plates incubated for further 4 d. Finally, 50 µl of 2.5× CPRG/Nonidet solution was added to all wells. A color reaction was visible within 2-6 h, which was quantified in an absorbance reader at 540 nm (Spectramax). Benznidazole was used as reference drug. Final in-test DMSO concentration did not exceed 1%. All assays were performed in two independent replicates at least.

### Activity Testing Against *Plasmodium falciparum*

*In vitro* antimalarial activity was tested against the erythrocytic stages of *P. falciparum* NF54, originally isolated from a patient at Schiphol airport. The parasites were grown in human erythrocytes in RPMI 1640 supplemented with 0.5% ALBUMAX^®^ II, 25 mM Hepes, 25 mM NaHCO_3_ (pH 7.3), 0.36 mM hypoxanthine, and 100 U/ml neomycin and kept in an atmosphere of 3% O_2_, 4% CO_2_, and 93% N_2_ in humidified modular chambers at 37°C. In the two-concentration assay, 100 µl medium containing the corresponding samples concentration (final sample concentration of 10 or 2 µg/ml) was added to the wells of a 96-well plate. For the IC_50_ determination, a 50-µl medium was added to each well and a serial sample dilution of 11 threefold dilution steps covering a final range from 100 to 0.002 μg/ml were prepared. Then 100-µl parasite (erythrocytes at 1.25% final hematocrit and 0.3% final parasitemia) was added. After 48 h of incubation with test compounds, 0.25 μCi of [^3^H]hypoxanthine was added per well, and the plates were incubated for an additional 24 h. Cells were harvested onto glass-fiber filters, and radioactivity was counted using a Betaplate liquid scintillation counter. Artemisinin was used as reference drug. Final in-test DMSO concentration did not exceed 1%. All assays were performed in two independent replicates at least.

### Clustering According to Antiprotozoal Activity

Two-way clustering was performed on the bioactivity data measured at 2 µg/ml (**Supplementary Table S1**). Percent inhibition was converted to decimals, and the maximum was set to 1. For sake of clarity, we included only one fraction per plant, i.e. the one which had exhibited the highest activity against any of the four protozoan parasites. Hierarchical clustering was performed with the Eisen lab programs *Cluster* and *Treeview* (Eisen et al., 1998) using Euclidean distance and average linkage.

### Cytotoxicity Testing

L6 rat skeletal myoblast cells were seeded in 96-well microtiter plates at 2 × 10^4^ cells/ml in RPMI 1640 medium supplemented with 10% FBS and 1.7 µM l-glutamine. The cells were allowed to attach overnight, then test compounds were added. After 72 h of incubation, 10 µl of resazurin solution (see above) was added, and the plates were incubated for an additional 2 h. Plates were read in a fluorescence scanner at 536 nm excitation and 588 nm emission wavelength. Podophyllotoxin was used as reference. All assays were performed in two independent replicates at least.

## Results

### Review of Medicinal Plants From Sudan

Ethnopharmacological literature review based on scholarly databases (Pubmed, Medline, SciFinder) and other supporting documents revealed that 34 of the 62 plants had been recorded for use against leishmaniasis, trypanosomiasis or malaria, including the symptoms related to any of these diseases (**Table 1**). Several of the plants had also been investigated pharmacologically and had exhibited anti-infective activity (**Table 2**).

**Table 1:** Plants investigated in the present study that have a reported use as anti-infective in traditional medicine.

Plant species	Family	Vernacular name	Plant part	Traditional medicinal use
*Abutilon pannosum* var. *figarianum* (Webb) Verdc. (syn. *Abutilon figarianum* Webb.)	Malvaceae	Humbuk, Gargadan	Leaves	Malaria, hepatoprotective, antibacterial (Mohamed et al., 2010)
*Ambrosia artemisiifolia* L. (syn. *Ambrosia maritima* L.)	Asteraceae	Damsissa	Leaves	Malaria, kidney stones, renal colic, hypertension (Mahmoud et al., 1999)
*Anethum graveolens* L.	Apiaceae	Shabat, Dill	Fruit, seeds, oil	Colic, carminative, flatulence, and dyspepsia, joint swelling, sedative for babies, lactogenic (Jana and Shekhawat, 2010)
*Annona muricata* L.	Annonaceae		Leaves	Antitumor, antiparasitic (Moghadamtousi et al., 2015)
*Argemone mexicana* L.	Papaveraceae		Leaves	Malaria, early-stage trypansomiasis (Chibale et al., 2012)
*Aristolochia bracteolata* Lam.	Aristolochiaceae	Irg el Agrrab, Um Galagil	Root	Malaria, scorpion stings (Suleiman, 2015)
*Azadirachta indica* A.Juss.	Meliaceae	Neem	Oil	Malaria, antihelminthic (El-Tahir et al., 1999a)
*Boswellia papyrifera* (Caill. ex Delile) Hochst	Burseraceae	Luban	Gum	Cough, respiratory infections (Yagi et al., 2016)
*Cardiospermum halicacabum* L.	Sapindaceae		Leaves	Malaria, antiparasitic (Waako et al., 2005)
*Combretum glutinosum* Perr. ex DC.	Combretaceae	Habeil	Seeds	Fever, rheumatism (Traore et al., 2014)
*Combretum hartmannianum* Schweinf.	Combretaceae		Wood	Jaundice, diabetes, rheuma, wound healing, anthelminthic (Khalid et al., 2012)
*Croton gratissimus* var. *gratissimus* (syn. *Croton zambesicus* Müll.Arg.)	Euphorbiaceae	Um-Geleigla	Fruit	Malaria, hypertension, menstrual pain (Mohamed and Khan, 2009)
*Cymbopogon citratus* (DC.) Stapf	Poaceae	Lemon grass	Leaves	Kidney stones and infections, malaria (Dike et al., 2012)
*Cyperus rotundus* L.	Cyperaceae		Rhizome	Fever, stomach disorders, bowel irritation (Kabbashi et al., 2015)
*Grewia tenax (*Forssk.) Fiori	Tiliaceae	Godeim	Fruits	Malaria, iron deficiency (Gebauer et al., 2007)
*Guiera senegalensis* J.F.Gmel.	Combretaceae	Gubeish	Leaves	Jaundice, malaria, hyperglycemia (Suleiman, 2015)
*Haplophyllum tuberculatum* (Forssk.) A.Juss.	Rutaceae	Haza	Leaves	Malaria, asthma, kidney diseases, gynecological, and bowel disorders (Ahmed et al., 2010; Khalid et al., 2012)
*Jatropha curcas* L.	Euphorbiaceae	Habat El Muluk	Leaves	Malaria (O. Abiodun et al., 2011)
*Lupinus albus* subsp. *graecus* (Boiss. & Spruner) Franco & P.Silva (syn. *Lupinus termis* Forssk.	Leguminosae	Tormos	Seeds	Paste for eczema and herpes zoster (Antoun and Taha, 1981)
*Moringa oleifera* Lam.	Moringaceae	Shagarat al Rawag	Leaves	Antimicrobial, antipyretic, antihypertensive, antispasmodic, antiinflammatory (Ali et al., 2002; Anwar et al., 2007)
*Nauclea latifolia* Sm.	Rubiaceae	Karmadoda	Fruit, root bark	Malaria, abdominal disease, antimicrobial (Benoit-Vical et al., 1998; Alamin et al., 2015)
*Piper cubeba* L. f.	Piperaceae		Fruits	Respiratory and intestinal disorders, nephroprotective, anticancer, antimicrobial (Salehi et al., 2019)
*Prosopis chilensis* (Molina) Stuntz	Leguminosae	Miskeet	Leaves	Antiinflammatory, analgesic (Abodola et al., 2015)
*Senna occidentalis* (L.) Link (syn. *Cassia occidentalis* L.)	Leguminosae	Soreib	Aerial part	Malaria, jaundice (Suleiman, 2015)
*Striga hermonthica* (Delile) Benth.	Orobanchaceae	Al-buda	Stem	Malaria (Okpako and Ajaiyeoba, 2004)
*Tephrosia apollinea* (Delile) DC	Leguminosae	Dhawasi; Dhafra	Leaves	Antiangiogenic, antioxidant antiproliferative, anticancer (Hassan et al., 2014)
*Terminalia laxiflora* Engl.	Combretaceae	Darout	Bark	Fever and respiratory infections (Salih et al., 2018)
*Terminalia leiocarpa* (DC.) Baill. (syn. *Anogeissus leiocarpa* (DC.) Guill. & Perr.)	Combretaceae	Sahab	Bark	Cough, dysentry, giardiasis (Musa et al., n.d.)
*Typha angustifolia* L.	Typhaceae	Si’da	Stem	Leprosy wound bleeding, diarrhoea, anthelminthic, diuretic (Varpe et al., 2012)
*Vachellia nilotica* (L.) P.J.H.Hurter & Mabb. (syn. *Acacia nilotica* (L.) Delile)	Fabaceae	Sunt	Leaves	Malaria (El-Tahir et al., 1999b), respiratory infections, diarrhoea, haemorrhage (Clarkson et al., 2004)
*Xanthium strumarium* subsp. *brasilicum* (Vell.) O.Bolòs & Vigo (syn. *Xanthium brasilicum* Vell.)	Compositae		Leaves	Malaria (Chandel et al., 2012)
*Tinospora bakis* (A.Rich.) Miers	Menispermaceae	Irg alhagar	Root	Fever, diarrhoea, abdominal pain (Ahmed et al., 2010)
*Ziziphus spina-christi* (L.) Desf.	Rhamnaceae	Sidir	Leaves	Fever, spasmolytic, and anti- diarrhea (Khalid et al., 2012)

**Table 2:** Plants investigated in the present study for which anti-infective properties have been examined experimentally.

Plant species	Part	Tested activities	IC _50_ value	Active metabolite(s)	Ref
*Anethum graveolens* L.	Leaves	Antiplasmodial	–	Volatile oils	(Chibale et al., 2012)
*Annona muricata* L.	Leaves	Antileishmanial	25 µg/ml	Acetogenins	(Osorio et al., 2007)
*Argemone mexicana* L.	Leaves	Antiplasmodial	1.7 μg/ml	Protopine, allocryptopine, and berberine	(Simoes-Pires et al., 2014)
*Aristolochia bracteolata* Lam.	Root	Antiplasmodial	< 5 μg/ml	–	(El-Tahir et al., 1999b)
*Azadirachta indica* A.Juss.	Leaves	Antiplasmodial	2.5 μg/ml	Gedunin	(Khalid et al., 1989a; MacKinnon et al., 1997)
*Cardiospermum halicacabum* L.	Leaves	Antiplasmodial	42 µg/ml	–	(Kaushik et al., 2015)
					
*Combretum glutinosum* Perr. ex DC.	Leaves	Trypanocidal	26.5 µg/ml	–	(Traore et al., 2014)
*Combretum hartmannianum* Schweinf.	Bark	Antiplasmodial	0.2 μg/ml	–	(Ali et al., 2002)
*Commiphora myrrha* (Nees) Engl.	Gum resin	Trypanocidal	8.1 μg/ml	–	(Okba et al., 2018)
*Croton gratissimus* var. *gratissimus* (syn. *Croton zambesicus* Müll.Arg.)	Root	Antiplasmodial	–	Sesquiterpenes, monoterpenes, and alkaloids	(Okokon and Nwafor, 2009)
*Curcuma longa* L.	Rhizome	Antiplasmodial	3- 4.2 μg/ml	Curcumin, demethoxycurcumin, and bis- demethoxycurcumin.	(Rasmussen et al., 2000)
*Cymbopogon citratus* (DC.) Stapf	Leaves	Antiplasmodial	–	Essential oils	(Tchoumbougnang et al., 2005)
*Cyperus rotundus* L.	Whole plant	Antiplasmodial	–	Terpenes, monoterpenes, and sesquiterpenes.	(Peerzada et al., 2015)
*Guiera senegalensis* J.F.Gmel.	Leaves and roots	Antiplasmodial	4.08 µM	Guiranone A	(Silva and Gomes, 2003)
*Haplophyllum tuberculatum* (Forssk.) A.Juss.	Leaves	(1) Antileishmanial (2) Trypanocidal	(1) 16.59 μg/ml and (2) 0.2 μg/ml	(1) R-(+)-limonene (2) Justicidin B	(Gertsch et al., 2003; Hemmati and Seradj, 2016; Hamdi et al., 2018)
*Jatropha curcas* L.	Seeds	Trypanocidal	1.9 µg/ml *(T. brucei) and* 7.4 µg/ml, (*T. cruzi)*	Phorbol esters	(Khalid, 2012)
*Mangifera indica* L.	Stem bark	Antiplasmodial	>50 µg/ml	–	(Zirihi et al., 2005)
*Moringa oleifera* Lam.	Leaves	Antileishmanial	5.25 µM	Niazinin	(Kaur et al., 2014)
*Nauclea latifolia* Sm.	Stem and root	Antiplasmodial	0.9-3 µg/ml	Alkaloids tetrahydrodesoxycordifoline and 19-O-methylangustoline	(Benoit-Vical et al., 1998; Boucherle et al., 2016)
*Piper cubeba* L. f.	Fruits	*Antitrypanosomal against T. cruzi* amastigotes	87.9 µg/ml	Essential oil	(Esperandim et al., 2013)
*Senna occidentalis* (L.) Link (syn. *Cassia occidentalis* L.)	Leaves	Antiplasmodial	<3 µg/ml	Anthraquinones, terpenes, and flavonoids.	(Tona et al., 2004)
*Striga hermonthica* (Delile) Benth.	Whole plant	Antiplasmodial	274.8 µg/ml	–	(Okpako and Ajaiyeoba, 2004)
*Terminalia leiocarpa* (DC.) Baill. (syn. *Anogeissus leiocarpa* (DC.) Guill. & Perr.)	Bark	Antiplasmodial	19 µg/ml	Ellagic acid, gallic acid, and gentisic acid	(Ndjonka et al., 2012)
*Tinospora bakis* (A.Rich.) Miers	Roots	Antiplasmodial	28.6 µg/ml	Alkaloids	(Ouattara et al., 2006)
*Vachellia nilotica* (L.) P.J.H.Hurter & Mabb. (syn. *Acacia nilotica* (L.) Delile)	Seed	Antiplasmodial	1.5 µg/ml	Terpenoids and tannins.	(El-Tahir et al., 1999b)
*Xanthium strumarium* subsp. *brasilicum* (Vell.) O.Bolòs & Vigo (syn. *Xanthium brasilicum* Vell.)	Aerial parts	Antiplasmodial, Antitrypanosomal	0.09 µg/ml *(T. brucei)*, 2.95 µg/ml (*T. cruzi)*, 0.16 µg/ml (*L. donovani*), and 1.71 µg/ml (*P.falciparum*)	8-Epixanthatin 1beta,5beta-epoxide	(Nour et al., 2009)
*Ziziphus spina*-*christi* (L.) Desf.	Leaves	Antileishmanial	>30 µg/ml	–	(Ali et al., 2002)

### Testing for Antiparasitic Activity

The original extracts and all fractions obtained by partitioning were tested at two concentrations, 2 and 10 μg/ml, against the following panel of protozoan parasites: *T. b. rhodesiense* bloodstream form, *T. cruzi* intracellular amastigote form grown in rat L6 cells, *L. donovani* axenic amastigote form grown at low pH, and *P. falciparum* erythrocytic stage grown in human erythrocytes. Percent inhibition was calculated in comparison to untreated controls. All tests were carried out in independent duplicates. The results are compiled in **Supplementary Table S1**.

**Figure 1:**
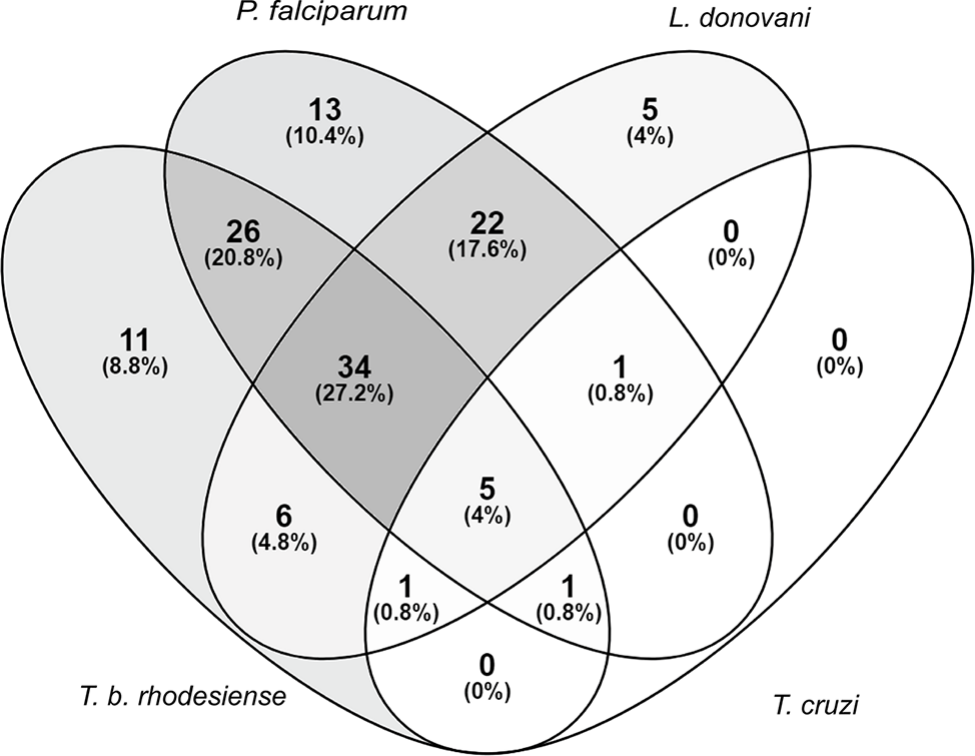
Susceptibility of parasites against a library of Sudanese medicinal plants. Activity criteria: >80% growth inhibition at 10 μg/ml or >50% growth inhibition at 2 μg/ml against one or more of the four included parasites. Venn diagram drawn with (https://bioinfogp.cnb.csic.es/tools/venny/).

Extracts that exhibited >80% growth inhibition at 10 μg/ml, or >50% growth inhibition at 2 μg/ml against at least one of the tested parasites was considered active. Of the 235 extracts in our library, 125 (53%) fulfilled these activity criteria. A total of 34 (27%) of the active extracts exhibited activity against *T. b. rhodesiense*, *L. donovani*, and *P. falciparum* collectively. Regarding parasite species-selective inhibition, *P. falciparum* appeared to be the most susceptible parasite, followed by *T. b. rhodesiense* and *L. donovani*. Among the tested parasites *T. cruzi* was the least susceptible towards the plant extracts (**Figure 1**).

### Two-Way Clustering of the Bioactivity Data

**Figure 2:**
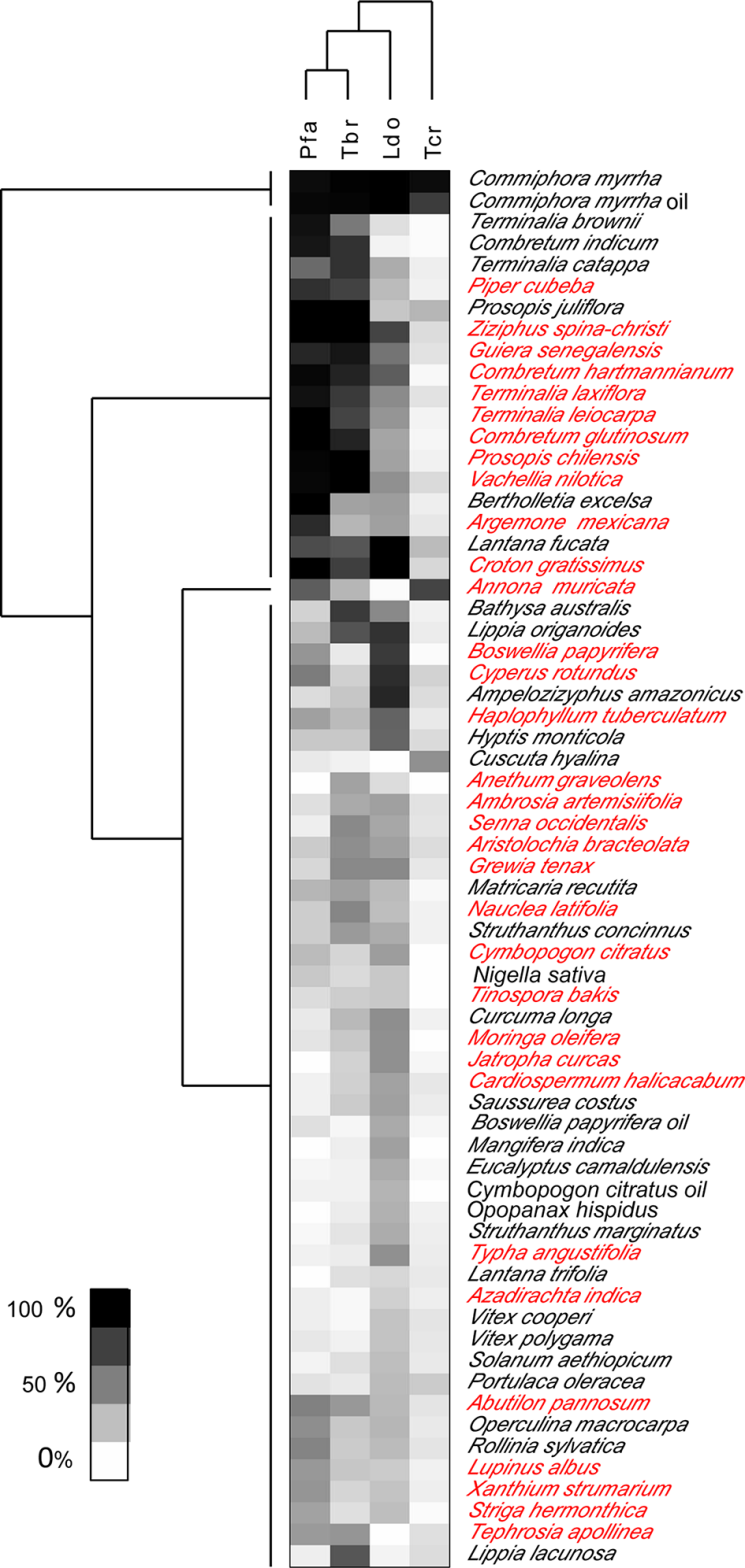
Heat map showing two-way clustering of bioactivity of extracts, and of parasites.

We used the screening results obtained with 2 µg/ml for two-way clustering, i.e. clustering the plants according to their bioactivity, and clustering the parasites according to their susceptibility (**Figure 2**). Per plant only one fraction from the partitioning was included, i.e. the one which had displayed the highest activity against any of the four parasites. This approach clearly confirmed the notion that *T. b. rhodesiense* and *P. falciparum*, despite their large phylogenetic distance, have a similar susceptibility profile. It also highlighted *T. cruzi* as the least susceptible of the four tested parasites (**Figure 2**). There was no clear separation between the medicinal plants with reported anti-infective use (printed in red in **Figure 2**) and the rest. Regarding antiplasmodial activity, the plants that had a reported use against malaria (n=17; **Table 1**) were slightly more active against *P. falciparum in vitro*, both at 2 µg/ml (mean inhibition of 43% vs. 39%) and at 10 µg/ml (mean inhibition of 89% vs. 75%). However, these differences were not statistically significant (p=0.70, two-tailed Mann-Whitney test).

### Testing for Cytotoxicity

Extracts with antiparasitic activity were also tested for cytotoxicity. This was done against rat L6 skeletal myoblast cells, the same cell line that had been used as host cells for testing against amastigote *T. cruzi*. Concentration-response curves allowed the calculation of both 50% and 90% inhibitory concentrations (IC_50_ and IC_90_; **Table 3**). The cytotoxicity data of the tested fractions cannot directly be compared to their antiparasitic activity because the antiparasitic and cytotoxic activity of a given fraction can be due to different molecules. Nevertheless, the aim was to identify non-toxic fractions for the following HPLC-based activity profiling and identification of active compounds.

**Table 3:** Cytotoxicity of antiprotozoal extracts as determined against rat L6 skeletal myoblast cells *in vitro*.

Plant	Part	Fraction	Cytotoxicity [µg/ml]	
			IC_50_	IC_90_
*Ambrosia artemisiifolia* L. (syn. *Ambrosia maritima* (L.))	Leaves	Ethyl acetate	38.1	85.8
*Annona muricata* L.	Leaves	Chloroform	20.3	71.3
*Argemone mexicana* L.	Leaves	Ethyl acetate	58.5	91.9
*Boswellia papyrifera* (Caill. ex Delile) Hochst	Gum	Petroleum ether	31.6	83.5
*Commiphora myrrha* (T.Nees) Engl.	Gum	Methanol	5.5	9.9
*Croton gratissimus* var. *gratissimus* (syn. *Croton zambesicus* Müll.Arg.)	Fruits	Chloroform	32.5	81.8
*Cuscuta hyalina* Roth ex Schult.	Stem	Chloroform	19.6	30.2
*Cymbopogon citratus* (DC.) Stapf	Leaves	Ethyl acetate	53.8	N/A^a^
*Cyperus rotundus* L.	Rhizome	Ethyl acetate	64.3	N/A^a^
*Guiera senegalensis* J.F.Gmel.	Leaves	Ethyl acetate	16.0	67.8
*Haplophyllum tuberculatum* (Forssk.) A.Juss.	Root	Chloroform	6.3	10.3
*Moringa oleifera* Lam.	Leaves	Ethyl acetate	89.6	N/A^a^
*Prosopis chilensis* (Molina) Stuntz	Leaves	Chloroform	5.9	9.8
*Struthanthus concinnus* Mart.	Branches	Ethyl acetate	44.6	86.1
*Tephrosia apollinea* (Delile) DC	Leaves	Chloroform	15.5	51.8
*Vachellia nilotica* (L.) P.J.H.Hurter & Mabb. (syn. *Acacia nilotica* (L.) Delile	Leaves	Ethyl acetate	21.5	83.0
*Xanthium strumarium* subsp. *brasilicum* (Vell.) O.Bolòs & Vigo (syn. *Xanthium brasilicum* Vell.)	Leaves	Petroleum ether	13.3	28.8

a N/A, not achievable.

### Extracts With Selective Anti-Trypanosomatid Activity

The most potent and selective activity against *T. b. rhodesiense* was exhibited by the chloroform fraction of the leaves of *Terminalia catappa* L. (Combretaceae), which showed 98% inhibition at 10 µg/ml and 80% inhibition at 2 µg/ml. Five of the ethyl acetate fractions showed growth inhibition > 85% at 10 µg/ml: fruits of *Croton gratissimus* var. *gratissimus* (syn. *Croton zambesicus* Muell. Arg. (Euphorbiaceae), processed fruits of *Nauclea latifolia* Sm. *(*Rubiaceae), leaves of *Lippia lacunosa* Mart. & Schauer (Verbenaceae), and *Xanthium strumarium* subsp. *brasilicum* (Vell.) O.Bolòs & Vigo (syn. *Xanthium brasilicum* Vell.) (Compositae), and the mango *Mangifera indica* L. fruit peels (Anacardiaceae). In addition, the water fraction of processed fruits of *Nauclea latifolia*. showed significant inhibition of *T. b. rhodesiense* at the two tested concentrations.

Only five percent of the library extracts were preferentially active against *L. donovani*. These were mostly lipophilic, e.g., the chloroform fraction of *Ambrosia artemisiifolia* L. (syn. *Ambrosia maritima* L.) (Asteraceae) leaves and the petroleum ether fractions of *Piper cubeba* L. f. (Piperaceae) fruits, *Portulaca oleracea* L. (Portulacaceae) aerial parts, and *Typha angustifolia* L. (Typhaceae) stem.

*Trypanosoma cruzi* was the least sensitive among the tested parasites. Only the crude extract of *Annona muricata* L. (Annonaceae) leaves and the methanolic fraction of *Commiphora myrrha* (Nees) Engl. (Burseraceae) oil and resin inhibited the growth of intracellular *T. cruzi* more than 50% at 2 µg/ml. However, these activities were not specific for *T. cruzi* (**Figure 1**, **Supplementary Table S1**).

### Extracts of Selective Antiplasmodial Activity

Thirteen fractions showed >80% growth inhibition of *P. falciparum* at 10 µg/ml, but none showed >50% growth inhibition at 2 µg/ml. Among the most active ones were the chloroform fraction of *Cuscuta hyalina* Roth ex Schult.(Convolvulaceae) stem and the ethyl acetate fractions of the leaves of *Abutilon pannosum* var. *figarianum* (Webb) Verdc. (syn. *Abutilon figarianum* Webb) (Malvaceae), *Annona muricata*, *Tephrosia apollinea* (Delile) DC (Leguminosae), and *Cardiospermum halicacabum* L. Moreover, both the chloroform and the ethyl acetate fractions of the leaves of *Cymbopogon citratus* (DC.) Stapf (Poaceae) exhibited selective antiplasmodial activity above 80% inhibition at 10 µg/ml. However, the ethyl acetate fraction, in particular, exhibited cytotoxicity on L6 cells with an IC_50_ of 53.8 µg/ml (**Table 3**).

### HPLC-Based Activity Profiling

**Figure 3:**
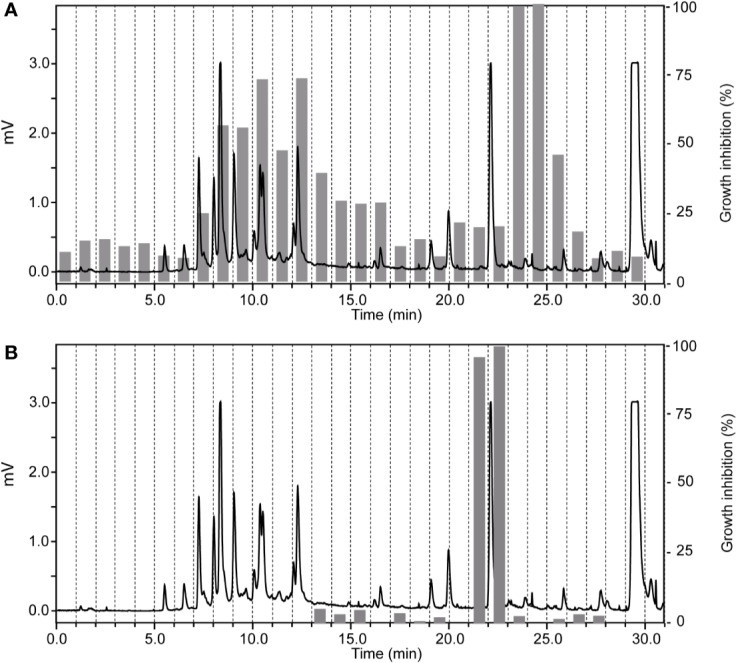
HPLC-based activity profiling of an ethyl acetate fraction from leaves of *Ziziphus spina-christi* (L.) Desf. The ELSD chromatogram of the fraction separation on an analytical RP-HPLC column is shown. Activity of the 1-min micro-fractions is indicated for trypanocidal **(A)** and antileishmanial activity **(B)**, expressed as % of growth inhibition.

The ethyl acetate fraction of *Ziziphus spina-christi* (L.) Desf. (Rhamnaceae) leaves had shown >80% growth inhibition at 10 µg/ml, and >50% inhibition at 2 µg/ml across all parasites (**Supplementary Table S1**). HPLC-based activity profiling revealed that the time-windows of antiparasitic activity against *L. donovani* on the one side, and against *T. b. rhodesiense* and *P. falciparum* on the other side, were different. The antitrypanosomal and antiplasmodial activity was associated with more polar, earlier eluting compounds, while the antileishmanial activity was located in the more lipophilic and later eluting compounds (**Figure 3**).

**Figure 4:**
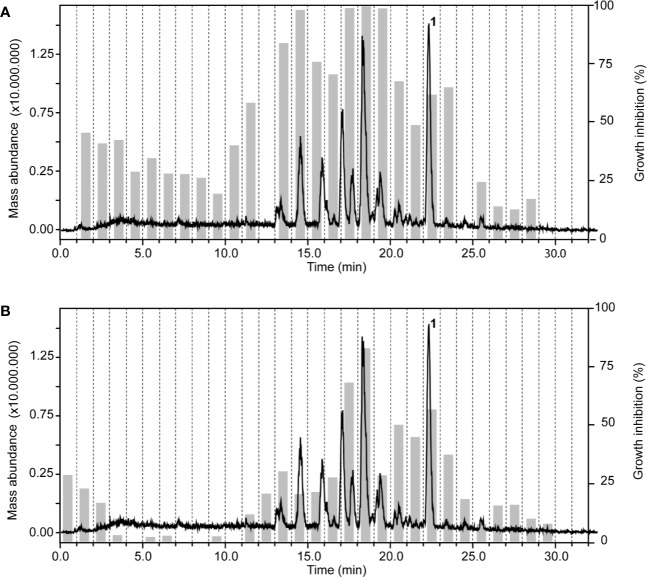
HPLC-ESIMS (base peak chromatogram) and activity profile of a chloroform fraction from leaves of *Guiera senegalensis* J.F.Gmel. Similar time window for trypanocidal **(A)** and antiplasmodial activity **(B)** was found. Peak **1** refers to guieranone A.

In the chloroform fraction of *Guiera senegalensis* J.F.Gmel. (Combretaceae) leaves the two time windows of activity against *T. b. rhodesiense* and *P. falciparum* were identical (**Figure 4**), likely indicating molecules of dual activity. However, the chloroform fraction also had a relatively high cytotoxicity (IC_50_ = 16 µg/ml; **Table 3**).

### Dereplication of Active Principles

HPLC-based activity profiling, in combination with on-line spectroscopic data (MS and UV) and comparison with natural products databases was used to dereplicate known active compounds. The antiplasmodial activity of *Guiera senegalensis* J.F.Gmel was in accordance with previous reports. In the window of activity a HPLC peak was detected which exhibited a [M+H]^+^ ion at *m/z* 316 in the MS, and λ_max_ 241 and 276 nm in the UV spectrum. This peak was assigned to guieranone A (MW 316.35 g/mol), a compound previously reported from this species (Silva and Gomes, 2003). The chloroform fraction of *Tephrosia apollinea* (Delile) DC. leaves was active against three parasites (**Supplementary Table S1**), as well as cytotoxic in L6 cells (**Table 3**). In the window of activity a HPLC peak exhibiting a [M+H]^+^ ion at *m/z* 393 in the ESIMS, and λ_max_ 256 and 310 nm in the UV spectrum corresponded to pseudosemiglabrin, a major secondary metabolite in this plant (Waterman and Khalid, 1980), of known antioxidant and anti-inflammatory activity.

**Figure 5:**
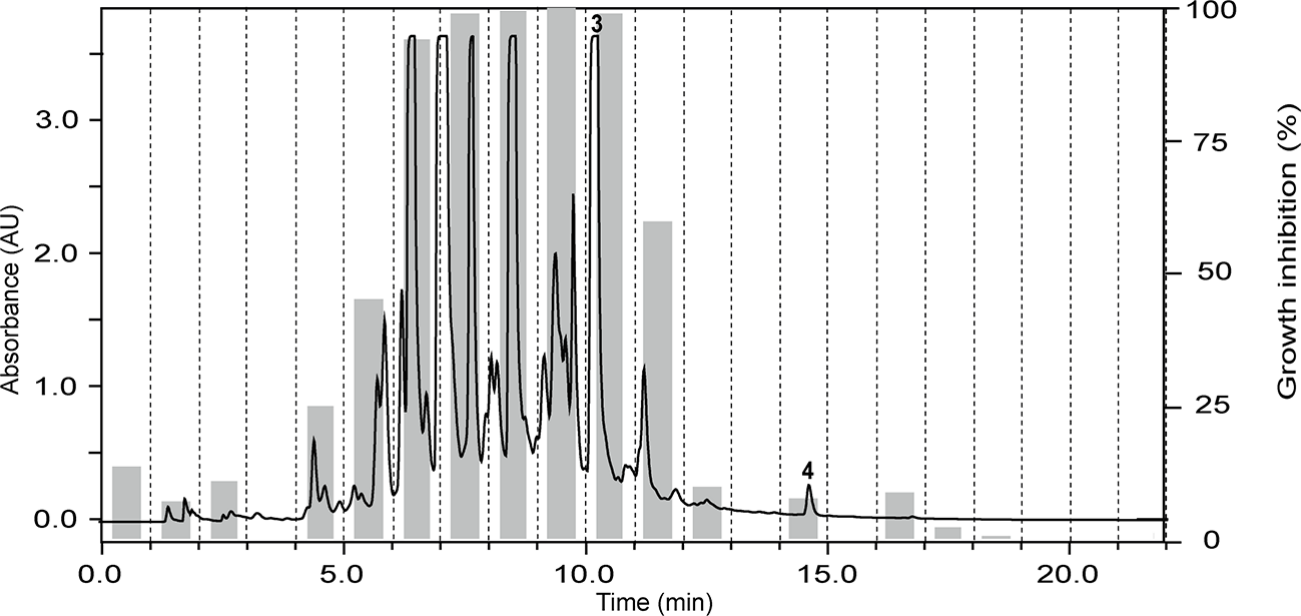
HPLC-PDA antiplasmodial activity profiling of the ethyl acetate fraction of the leaves of *Terminalia leiocarpa* (DC.) Baill. recorded at 254 nm. Peaks **3** and **4** refer to ellagic acid and quercetin, respectively.

**Figure 6:**
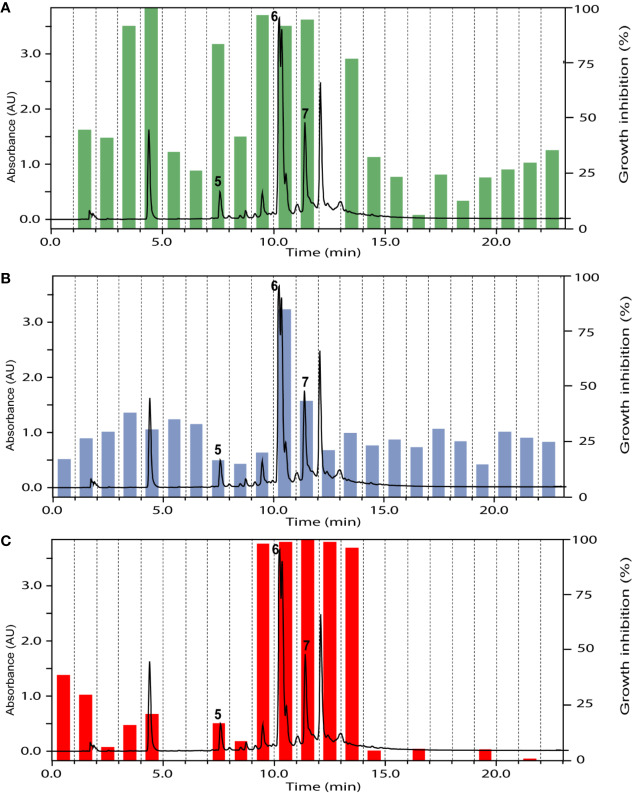
HPLC-UV trace of the ethyl acetate fraction of *Vachellia nilotica* (L.) P.J.H.Hurter & Mabb. leaves detected at 254 nm, and the % of inhibition of the 1-min micro-fractions against *Trypanosoma brucei rhodesiense* **(A)**, *Leishmania donovani* **(B)**, and *Plasmodium falciparum* **(C)**. Peaks **5** to **7** refer to catechin, ethyl gallate, and epicatechin gallate, respectively.

The ethyl acetate fraction of the leaves, roots, and seeds of *Terminalia leiocarpa* (DC.) Baill. (syn. *Anogeissus leiocarpa* (DC.) Guill. & Perr.) (Combretaceae) exhibited promising inhibitory activity against *T. b. rhodesiense* and *P. falciparum* (**Figure 5**). In the active time window HPLC peaks with MS and UV data indicative for ellagic acid and quercetin were seen, and their identity was confirmed by co-injection of authentic samples. The two compounds have been previously reported from *T. leiocarpa* (Ndjonka et al., 2012; Oboh et al., 2017). Ellagic acid has been previously shown to possess antiplasmodial activity (Banzouzi et al., 2002) which has been attributed to the inhibition of beta-haematin formation in the parasite (Dell’Agli et al., 2003). The antiplasmodial activity of quercetin (Ganesh et al., 2012) has been associated with the inhibition of a parasite protein kinase (Wiser et al., 1983). The leaf extract of *Vachellia nilotica* (L.) P.J.H.Hurter & Mabb. (syn. *Acacia nilotica* (L.) Delile) (Fabaceae) inhibited *T. b. rhodesiense* and *P. falciparum* at 2 µg/ml, and moderate cytotoxicity (IC_50_ of 21.5 µg/ml against L6; **Table 3**). In the HPLC activity profile (**Figure 6**) peaks with [M+H]^+^ ions at *m/z* 291.0 and *m/z* 442.9 in the ESIMS, and with λ_max_ 277 and 280 nm in the UV spectra were detected in the active time window. These peaks corresponded to catechin (Wulf et al., 2008) and epicatechin gallate (Salem et al., 2011), respectively. The occurrence of these compounds in *V. nilotica* has been reported (Khalid et al., 1989b; Dikti Vildina et al., 2017). Catechins were found to possess antiplasmodial activity by inhibiting both the ATPase and chaperone functions of the *P. falciparum* heat shock proteins (PfHsps) through direct binding to PfHsp70-1 and PfHsp70-z (Zininga et al., 2017). In addition, a peak corresponding to ethyl gallate was detected in the active time window. Gallate esters are known inhibitors of trypanosome alternative oxidase, and they can increase intracellular glycerol to toxic levels resulting in trypanocidal activity (Jeacock et al., 2017). However, we cannot exclude that ethyl gallate was formed from gallic acid during ethanol extraction.

## Discussion

A total of 62 Sudanese plants were selected on the basis of their traditional use as medicinal plants, with an emphasis on plants that had been used to treat protozoal diseases. Of these plants a library of 235 extracts was prepared and tested against four protozoan parasites: *Plasmodium falciparum* (erythrocytic stages), *Trypanosoma brucei rhodesiense* (bloodstream forms), *Trypanosoma cruzi* (intracellular amastigotes), and *Leishmania donovani* (axenic amastigotes). The methods used were standard *in vitro* tests for drug discovery, where the measured signals correlated with the number of parasites. Screening of the library resulted in 125 potential hits that fulfilled the chosen activity criteria, i.e. > 80% growth inhibition at 10 μg/ml or >50% growth inhibition at 2 μg/ml against one or more of the four parasites. A total of 11 extracts were solely active against *T. b. rhodesiense*, 13 against *P. falciparum*, and 5 against *L. donovani*. A total of 27 extracts exhibited activity against three parasites. The percentage of extracts that displayed activity against both *T. brucei* and *P. falciparum* (21%) was considerably higher than that with activity against *T. brucei* and *L. donovani* (5%), despite the fact that trypanosomes and leishmania are taxonomically related trypanosomatid parasites. This somehow surprising result is in agreement with previous screening campaigns reports (Mokoka et al., 2011; Kaiser et al., 2015; Llurba Montesino et al., 2015). The lack of overlap between activity against *T. cruzi* and *L. donovani* is not unusual and has been documented previously (Witschel et al., 2012; Zulfiqar et al., 2017). They are different parasites living in different compartments, i.e. cytoplasma for *T. cruzi* but acidic environment for *Leishmania*.

Interestingly, a major part of these extracts were from plants of the family Combretaceae (*Guiera senegalensis* J.F.Gmel*., T. leiocarpa* (DC.) Baill., *Combretum glutinosum* Perr. ex DC*., Combretum indicum* (L.) DeFilipps (syn. *Quisqualis indica* L.), and *Terminalia laxiflora* Engl.). Plants of this family are known to be rich in phenolic compounds. The lowest number of hits was found for *T. cruzi*. This may be due, in part, to the fact that *T. cruzi* amastigotes (which are the clinically relevant stages for chemotherapy) cannot be grown axenically. Hence, activity can only be identified if the antiparasitic activity against *T. cruzi* is significantly higher than cytotoxicity in L6 cells used for culturing the parasite.

**Figure 7:**
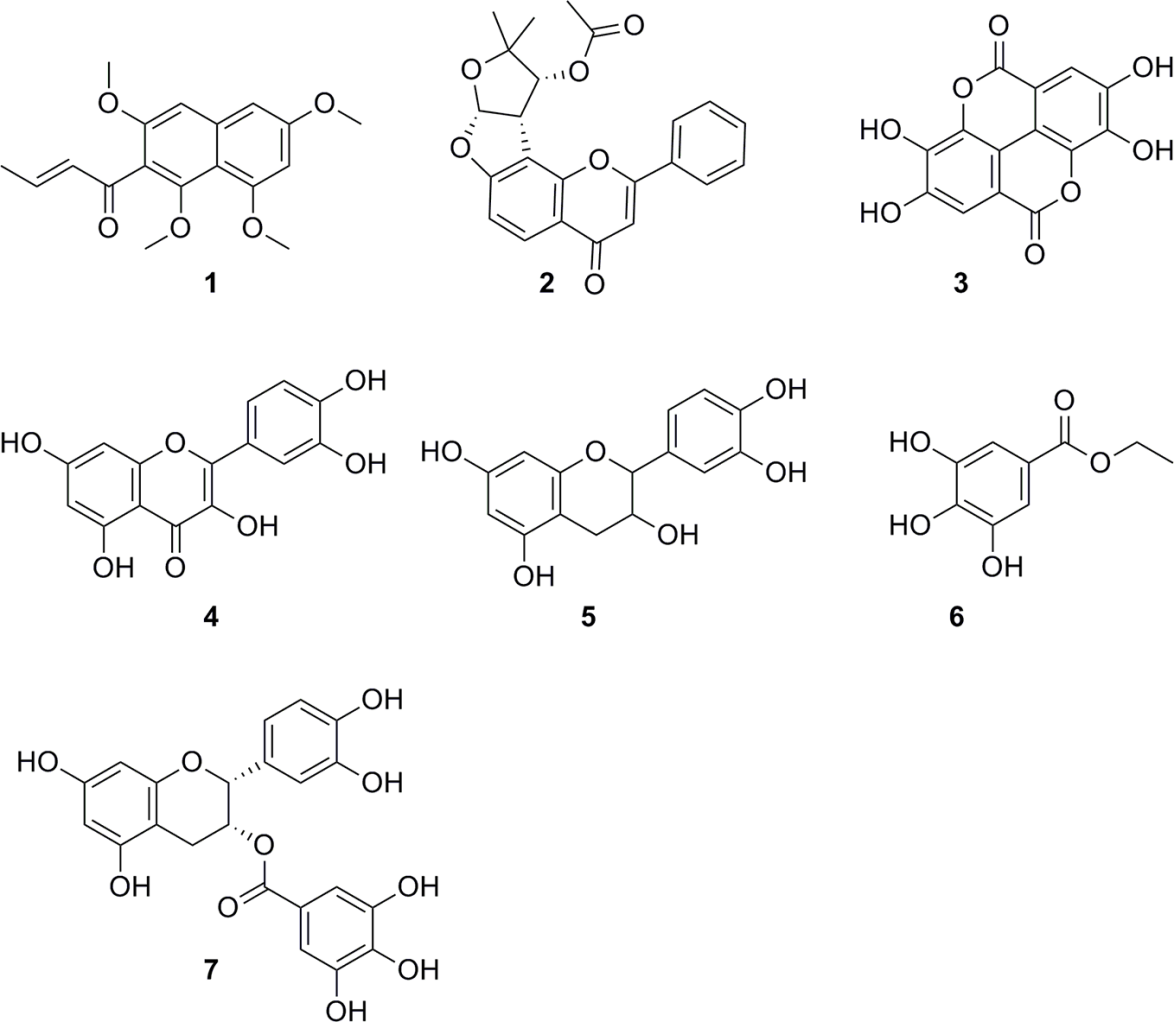
Chemical structures of compounds identified by dereplication. Guieranone A (**1**), pseudosemiglabrin (**2**), ellagic acid (**3**), quercetin (**4**), catechin (**5**), ethyl gallate (**6**), and epicatechin gallate (**7**).

Our findings corroborate previously reported activities of some plants, e.g. for *Z. spina-christi* (Mubaraki et al., 2017), *G. senegalensis* (Fiot et al., 2006), *Terminalia* spp. and *X. strumarium* (Nour et al., 2009; Abiodun et al., 2012; Ndjonka et al., 2012). Antiprotozoal activities of some other plants are reported here for the first time, e.g. the antitrypanosomal activity of *Cuscuta hyalina* Roth ex Schult.*, Combretum indicum* (L.) DeFilipps, and *Croton gratissimus* var. *gratissimus*. HPLC activity profiling, in combination with on-line spectroscopy, enabled a rapid identification of some of the active compounds by dereplication (**Figure 7**), i.e. guieranone A (**1**) from *G. senegalensis*, pseudosemiglabrin (**2**) from *T. apollinea*, ellagic acid (**3**), and quercetin (**4**) from *T. leiocarpa*, and catechin (**5**), ethyl gallate (**6**), and epicatechin gallate (**7**) from *V. nilotica*. HPLC-based activity profiling will also be of use for the identification of antiprotozoal compounds from promising Sudanese plants such as *Croton gratissimus* var. *gratissimus* and *Cuscuta hyalina* Roth ex Schult., which exhibited interesting antitrypanosomatid activity. In summary, we have compiled a comprehensive library of Sudanese medicinal plants and demonstrate that they are a promising source of bioactive molecules against protozoan parasites.

## Data Availability Statement

All datasets generated for this study are included in the article/**Supplementary Material**.

## Author Contributions

Conceived and designed the experiments: AM, PM, MK, MH, SK. Performed the experiments: AM, MK. Analyzed the data: PM, MH, SK. Wrote the paper: AM, PM, MK, MH, SK.

## Funding

This work was supported by grants to AM by the Amt für Ausbildungsbeiträge Basel (www.hochschulen.bs.ch/ueber-uns/organisation/amt-ausbildungsbeitraege.html) and the Emilia Guggenheim-Schnurr Foundation (www.ngib.ch/stiftung-egs). The funders had no role in study design, data collection and analysis, decision to publish, or preparation of the manuscript.

## Conflict of Interest

The authors declare that the research was conducted in the absence of any commercial or financial relationships that could be construed as a potential conflict of interest.

## Supplementary Material

Table S1 Screening results of protozoan activity of plant extracts at two concentrations, 10 and 2 µg/ml.

## References

[B1] Abiodun O. Gbotosho G. Ajaiyeoba E. Happi T. Falade M. Wittlin S. (2011). In Vitro Antiplasmodial Activity and Toxicity Assessment of Some Plants from Nigerian Ethnomedicine. Pharmaceut. Biol. 49 (1), 9–14. 10.3109/13880209.2010.490224 20738218

[B2] Abiodun O. O. Gbotosho G. O. Ajaiyeoba E. O. Brun R. Oduola A. M. (2012). Antitrypanosomal Activity of Some Medicinal Plants from Nigerian Ethnomedicine. Parasitol. Res. 110 (2), 521–526. 10.1007/s00436-011-2516-z 21789586

[B3] Abodola M. A. Lutfi M. F. Bakhiet A. O. Mohamed A. H. (2015). The Anti-Inflammatory and Analgesic Properties of Prosopis Chilenses in Rats. Int. J. Health Sci. 9 (3), 265–271. 10.12816/0024693 PMC4633190 26609291

[B4] Ahmed E. Nour B. Y. Mohammed Y. G. Khalid H. S. (2010). Antiplasmodial Activity of Some Medicinal Plants Used in Sudanese Folk-medicine. Environmental Health Insights 4, 1–6. 10.4137/ehi.s4108 20523878 PMC2879607

[B5] Alamin M. A. Yagi A. I. Yagi S. M. (2015). Evaluation of Antidiabetic Activity of Plants Used in Western Sudan. Asian Pacific J. Trop. Biomed. 5 (5), 395–402. 10.1016/S2221-1691(15)30375-0

[B6] Ali H. König G. M. Khalid S. A. Wright A. D. Kaminsky R. (2002). Evaluation of Selected Sudanese Medicinal Plants for Their in Vitro Activity against Hemoflagellates, Selected Bacteria, HIV-1-RT and Tyrosine Kinase Inhibitory, and for Cytotoxicity. J. Ethnopharmacol. 83 (3), 219–228. 10.1016/S0378-8741(02)00245-3 12426089

[B7] Antoun M. D. Taha O. M. (1981). Studies on Sudanese Medicinal Plants. II. Evaluation of an Extract of Lupinus Termis Seeds in Chronic Eczema. J. Natural Prod. 44 (2), 179–183. 10.1021/np50014a006 7017074

[B8] Anwar F. Latif S. Ashraf M. Gilani A. H. (2007). Moringa Oleifera: A Food Plant with Multiple Medicinal Uses. Phytother. Res.: PTR 21 (1), 17–25. 10.1002/ptr.2023 17089328

[B9] Ariey Frédéric Witkowski B. Amaratunga C. Beghain J. Langlois A.-C. Khim N. (2014). A Molecular Marker of Artemisinin-Resistant Plasmodium Falciparum Malaria. Nature 505 (7481), 50–55. 10.1038/nature12876 24352242 PMC5007947

[B10] Baltz T. Baltz D. Giroud C. Crockett J. (1985). Cultivation in a Semi-Defined Medium of Animal Infective Forms of Trypanosoma Brucei, T. Equiperdum, T. Evansi, T. Rhodesiense and T. Gambiense. EMBO J. 4 (5), 1273–1277. 10.1002/j.1460-2075.1985.tb03772.x 4006919 PMC554336

[B11] Banzouzi J.-T. Prado R. Menan H. Valentin A. Roumestan C. Mallie M. (2002). In Vitro Antiplasmodial Activity of Extracts of Alchornea Cordifolia and Identification of an Active Constituent: Ellagic Acid. J. Ethnopharmacol. 81 (3), 399–401. 10.1016/s0378-8741(02)00121-6 12127243

[B12] Benoit-Vical Françoise Valentin A. Cournac Valérie Pélissier Y. Mallié Michèle Bastide J.-M. (1998). In Vitro Antiplasmodial Activity of Stem and Root Extracts of Nauclea Latifolia S.M. (Rubiaceae). J. Ethnopharmacol. 61 (3), 173–178. 10.1016/S0378-8741(98)00036-1 9705007

[B13] Boucherle B. Haudecoeur R. Ferreira Queiroz E. De Waard M. Wolfender J.-L. Robins R. J. (2016). Nauclea Latifolia: Biological Activity and Alkaloid Phytochemistry of a West African Tree. Natural Prod. Rep. 33 (9), 1034–1043. 10.1039/C6NP00039H 27346294

[B14] Buckner F. S. Verlinde C. L. La Flamme A. C. Van Voorhis W. C. (1996). Efficient Technique for Screening Drugs for Activity against Trypanosoma Cruzi Using Parasites Expressing Beta-Galactosidase. Antimicrobial Agents Chemother. 40 (11), 2592–2597. 10.1128/AAC.40.11.2592 PMC163582 8913471

[B15] Chandel S. Bagai U. Vashishat N. (2012). Antiplasmodial Activity of Xanthium Strumarium against Plasmodium Berghei-Infected BALB/c Mice. Parasitol. Res. 110 (3), 1179–1183. 10.1007/s00436-011-2611-1 21847597

[B16] Chibale K. Davies-Coleman M. T. Collen Mutowembwa Masimirembwa (2012). Drug Discovery in Africa: Impacts of Genomics, Natural Products, Traditional Medicines, Insights into Medicinal Chemistry, and Technology Platforms in Pursuit of New Drugs. (Heidelberg: Springer).

[B17] Clarkson C. Maharaj V. J. Crouch N. R. Grace O. M. Pillay P. Matsabisa M. G. (2004). In Vitro Antiplasmodial Activity of Medicinal Plants Native to or Naturalised in South Africa. J. Ethnopharmacol. 92 (2–3), 177–191. 10.1016/j.jep.2004.02.011 15137999

[B18] Dell’Agli M. Parapini S. Basilico N. Verotta L. Taramelli D. Berry C. (2003). In Vitro Studies on the Mechanism of Action of Two Compounds with Antiplasmodial Activity: Ellagic Acid and 3,4,5-Trimethoxyphenyl(6′-O-Aalloyl)-Beta-D-Glucopyranoside. Planta Med. 69 (2), 162–164. 10.1055/s-2003-37706 12624824

[B19] Dike I.P. Olawole Obembe O. Adebiyi F.E. (2012). Ethnobotanical Survey for Potential Anti-Malarial Plants in South-Western Nigeria. J. Ethnopharmacol. 144 (3), 618–626. 10.1016/j.jep.2012.10.002 23085021

[B20] Dikti Vildina J. Kalmobe J. Djafsia B. Schmidt T. J. Liebau E. Ndjonka D. (2017). Anti-Onchocerca and Anti-Caenorhabditis Activity of a Hydro-Alcoholic Extract from the Fruits of Acacia Nilotica and Some Proanthocyanidin Derivatives. Mol. (Basel Switzerland) 22 (5), 748. 10.3390/molecules22050748 PMC6154738 28481237

[B21] Eisen M. B. Spellman P. T. Brown P. O. Botstein D. (1998). Cluster Analysis and Display of Genome-Wide Expression Patterns. Proc. Natl. Acad. Sci. United States America 95 (25), 14863–14868. 10.1073/pnas.95.25.14863 PMC24541 9843981

[B22] El Tahir A. Satti G. M. H. Khalid S. A. (1999a). Antiplasmodial Activity of Selected Sudanese Medicinal Plants with Emphasis on Maytenus Senegalensis (Lam.) Exell. J. Ethnopharmacol. 64 (3), 227–233. 10.1016/S0378-8741(98)00129-9 10363837

[B23] El-Tahir A. Satti G. M. Khalid S. A. (1999b). Antiplasmodial Activity of Selected Sudanese Medicinal Plants with Emphasis on Acacia Nilotica. Phytother. Res.: PTR 13 (6), 474–478. 10.1002/(SICI)1099-1573(199909)13:6<474::AID-PTR482>3.0.CO;2-6 10479756

[B24] Esperandim V. R. da Silva Ferreira D. Cristina K. Rezende S. Guidi Magalhães L. Medeiros Souza J. (2013). In Vitro Antiparasitic Activity and Chemical Composition of the Essential Oil Obtained from the Fruits of Piper Cubeba. Planta Med. 79 (17), 1653–1655. 10.1055/s-0033-1351022 24288276

[B25] Feher M. Schmidt J. M. (2003). Property Distributions: Differences between Drugs, Natural Products, and Molecules from Combinatorial Chemistry. J. Chem. Inf. Comput. Sci. 43 (1), 218–227. 10.1021/ci0200467 12546556

[B26] Filardy A. A. Guimarães-Pinto K. Nunes M. P. Zukeram K. Fliess L. Pereira L. (2018). Human Kinetoplastid Protozoan Infections: Where Are We Going Next? Front. Immunol. 9, 1493. 10.3389/fimmu.2018.01493 30090098 PMC6069677

[B27] Fiot J. Sanon S. Azas N. Mahiou Valérie Jansen O. Angenot L. (2006). Phytochemical and Pharmacological Study of Roots and Leaves of Guiera Senegalensis J.F. Gmel (Combretaceae). J. Ethnopharmacol. 106 (2), 173–178. 10.1016/j.jep.2005.12.030 16443341

[B28] Ganesh D. Fuehrer H.-P. Starzengrüber P. Swoboda P. Khan W. A. Reismann J. A.B. (2012). Antiplasmodial Activity of Flavonol Quercetin and Its Analogues in Plasmodium Falciparum: Evidence from Clinical Isolates in Bangladesh and Standardized Parasite Clones. Parasitol. Res. 110 (6), 2289–2295. 10.1007/s00436-011-2763-z 22215188

[B29] Gebauer J. El-Siddig K. El Tahir B. A. Salih A. A. Ebert G. Hammer K. (2007). Exploiting the Potential of Indigenous Fruit Trees: Grewia Tenax (Forssk.) Fiori in Sudan. Genet. Resour. Crop Evol. 54 (8), 1701–1708. 10.1007/s10722-006-9178-1

[B30] Gertsch Jürg Tobler R. Thöni Brun R. Sticher O. Heilmann Jörg (2003). Antifungal, Antiprotozoal, Cytotoxic and Piscicidal Properties of Justicidin B and a New Arylnaphthalide Lignan from Phyllanthus piscatorum. Planta Med. 69 (5), 420–424. 10.1055/s-2003-39706 12802722

[B31] Hamdi A. Bero J. Beaufay C. Flamini G. Marzouk Z. Vander Heyden Y. (2018). In Vitro Antileishmanial and Cytotoxicity Activities of Essential Oils from Haplophyllum Tuberculatum A. Juss Leaves, Stems and Aerial Parts. BMC Complement. Altern. Med. 18 (1), 60. 10.1186/s12906-018-2128-6 29444667 PMC5813356

[B32] Hassan L. E. A. Ahamed M. B.K. Abdul Majid A. S. Baharetha H. M. Muslim N. S. Nassar Z. D. (2014). Correlation of Antiangiogenic, Antioxidant and Cytotoxic Activities of Some Sudanese Medicinal Plants with Phenolic and Flavonoid Contents. BMC Complement. Altern. Med. 14, 406. 10.1186/1472-6882-14-406 PMC4210631 25331269

[B33] Hemmati S. Seradj H. (2016). Justicidin B: A Promising Bioactive Lignan. Molecules 21 (7), 820. 10.3390/molecules21070820 PMC6272961 27347906

[B34] Hotez P. J. Kamath A. (2009). Neglected Tropical Diseases in Sub-Saharan Africa: Review of Their Prevalence, Distribution, and Disease Burden. PloS Neglected Trop. Dis. 3 (8), e412. 10.1371/journal.pntd.0000412 PMC2727001 19707588

[B35] Jana S. Shekhawat G. S. (2010). Anethum Graveolens: An Indian Traditional Medicinal Herb and Spice. Pharmacogn. Rev. 4 (8), 179–184. 10.4103/0973-7847.70915 22228959 PMC3249919

[B36] Jeacock L. Baker N. Wiedemar N. Mäser P. Horn D. (2017). Aquaglyceroporin-Null Trypanosomes Display Glycerol Transport Defects and Respiratory-Inhibitor Sensitivity. PloS Pathog. 13 (3), e1006307. 10.1371/journal.ppat.1006307 28358927 PMC5388498

[B37] Kabbashi A. S. Mohammed S. E. A. Almagboul A. Z. Ahmed I. F. (2015). Antimicrobial Activity and Cytotoxicity of Ethanolic Extract of Cyperus Rotundus L. Am. J. Pharm. Pharmaceut. Sci. 2 (1), 13.

[B38] Kaiser M. Maes L. Tadoori L. P. Spangenberg T. Ioset J.-R. (2015). Repurposing of the Open Access Malaria Box for Kinetoplastid Diseases Identifies Novel Active Scaffolds against Trypanosomatids. J. Biomol. Screen. 20 (5), 634–645. 10.1177/1087057115569155 25690568

[B39] Kaur A. Kaur P. K. Singh S. Singh I. P. (2014). "Antileishmanial Compounds from Moringa Oleifera Lam.” *Zeitschrift* Fur Naturforschung. C. J. Biosci. 69 (3–4), 110–116. 10.5560/znc.2013-0159 24873031

[B40] Kaushik N. K. Bagavan A. Rahuman A. A. Zahir A. A. Kamaraj C. Elango G. (2015). Evaluation of Antiplasmodial Activity of Medicinal Plants from North Indian Buchpora and South Indian Eastern Ghats. Malaria J. 14, 65. 10.1186/s12936-015-0564-z PMC4340493 25879738

[B41] Khalid S. A. Duddeck H. Gonzalez-Sierra M. (1989a). Isolation and Characterization of an Antimalarial Agent of the Neem Tree Azadirachta Indica. J. Natural Prod. 52 (5), 922–926. 10.1021/np50065a002 2607354

[B42] Khalid S. A. Yagi S. M. Khristova P. Duddeck H. (1989b). (+)-Catechin-5-Galloyl Ester as a Novel Natural Polyphenol from the Bark of Acacia Nilotica of Sudanese Origin1. Planta Med. 55 (6), 556–558. 10.1055/s-2006-962094 17262478

[B43] Khalid H. Abdalla W. E. Abdelgadir H. Opatz T. Efferth T. (2012). Gems from Traditional North-African Medicine: Medicinal and Aromatic Plants from Sudan. Natural Prod. Bioprospect. 2 (3), 92–103. 10.1007/s13659-012-0015-2

[B44] Khalid S. A. (2012). "Natural Product-Based Drug Discovery Against Neglected Diseases with Special Reference to African Natural Resources," in *Drug Discovery in Africa*. Eds. Chibale K. Davies-Coleman M. Masimirembwa C. (Berlin, Heidelberg: Springer). 10.1007/978-3-642-28175-4_9

[B45] Leishmaniasis (n.d). Accessed October 22, 2019 https://www.who.int/news-room/fact-sheets/detail/leishmaniasis.

[B46] Llurba Montesino Núria Kaiser M. Brun R. Schmidt T. J. (2015). Search for Antiprotozoal Activity in Herbal Medicinal Preparations; New Natural Leads against Neglected Tropical Diseases. Mol. (Basel Switzerland) 20 (8), 14118–14138. 10.3390/molecules200814118 PMC6332118 26248069

[B47] Lu F. Culleton R. Meihua Z. Ramaprasad A. von Seidlein L. Zhou H. (2017). Emergence of Indigenous Artemisinin-Resistant Plasmodium Falciparum in Africa. New Engl. J. Med. 376 (10), 991–993. 10.1056/NEJMc1612765 28225668

[B48] Ménard D. Khim N. Beghain J. Adegnika A. A. Shafiul-Alam M. Amodu O. (2016). A Worldwide Map of Plasmodium Falciparum K13-Propeller Polymorphisms. New Engl. J. Med. 374 (25), 2453–2464. 10.1056/NEJMoa1513137 27332904 PMC4955562

[B49] MacKinnon S. Durst T. Arnason J. T. Angerhofer C. Pezzuto J. Sanchez-Vindas P. E. (1997). Antimalarial Activity of Tropical Meliaceae Extracts and Gedunin Derivatives. J. Natural Prod. 60 (4), 336–341. 10.1021/np9605394 9134742

[B50] Mahmoud A. A. Ahmed A. A. Bassuony A. A. (1999). A New Chlorosesquiterpene Lactone from Ambrosia Maritima. Fitoterapia 70 (6), 575–578. 10.1016/S0367-326X(99)00091-X

[B51] Mesu V. K. B. Ku Kalonji W. M. Bardonneau Clélia Valverde Mordt O. Blesson Séverine Simon François (2018). Oral Fexinidazole for Late-Stage African Trypanosoma Brucei Gambiense Trypanosomiasis: A Pivotal Multicentre, Randomised, Non-Inferiority Trial. Lancet (London England) 391 (10116), 144–154. 10.1016/S0140-6736(17)32758-7 29113731

[B52] Mikus J. Steverding D. (2000). A Simple Colorimetric Method to Screen Drug Cytotoxicity against Leishmania Using the Dye Alamar Blue®. Parasitol. Int. 48 (3), 265–269. 10.1016/S1383-5769(99)00020-3 11227767

[B53] Moghadamtousi S. Z. Fadaeinasab M. Nikzad S. Mohan G. Ali H. M. Kadir H. A. (2015). Annona Muricata (Annonaceae): A Review of Its Traditional Uses, Isolated Acetogenins and Biological Activities. Int. J. Mol. Sci. 16 (7), 15625–15658. 10.3390/ijms160715625 26184167 PMC4519917

[B54] Mohamed I. E. Khan S. N. (2009). Bioactive Natural Products from Two Sudanese Medicinal Plants Diospyros Mespiliformis and Croton Zambesicus. Rec. Nat. Prod. 3(4), 198–203.

[B55] Mohamed I. El T. Nur El B. El S. Abdelrahman M. El N. (2010). The Antibacterial, Antiviral Activities and Phytochemical Screening of Some Sudanese Medicinal Plants. EurAsian J. Biosci. 4 (1), 8–16. 10.5053/ejobios.2010.4.0.2

[B56] Mokoka T. A. Zimmermann S. Julianti T. Hata Y. Moodley N. Cal M. (2011). In Vitro Screening of Traditional South African Malaria Remedies against Trypanosoma Brucei Rhodesiense, Trypanosoma Cruzi, Leishmania Donovani, and Plasmodium Falciparum. Planta Med. 77 (14), 1663–1667. 10.1055/s-0030-1270932 21412695

[B57] Mubaraki M. A. Hafiz T. A. Al-Quraishy S. Dkhil M. A. (2017). Oxidative Stress and Genes Regulation of Cerebral Malaria upon Zizyphus Spina-Christi Treatment in a Murine Model. Microbial. Pathogen. 107, 69–74. 10.1016/j.micpath.2017.03.017 28336326

[B58] Musa M. S. Abdelrasool F. E. Elsheikh E. A. Mahmoud A. L. E. Yagi S. M. (n.d). “Ethnobotanical Study of Medicinal Plants in the Blue Nile State, South-Eastern Sudan,” 11.

[B59] Ndjonka Dieudonné Bergmann Bärbel Agyare C. Zimbres FláviaM. Lüersen K. Hensel A. (2012). In Vitro Activity of Extracts and Isolated Polyphenols from West African Medicinal Plants against Plasmodium Falciparum. Parasitol. Res. 111 (2), 827–834. 10.1007/s00436-012-2905-y 22476602

[B60] Newman D. J. Cragg G. M. (2016). Natural Products as Sources of New Drugs from 1981 to 2014. J. Natural Prod. 79 (3), 629–661. 10.1021/acs.jnatprod.5b01055 26852623

[B61] Nour A. Khalid S. Kaiser M. Brun R. Abdallah Wai’l Schmidt T. (2009). “The Antiprotozoal Activity of Sixteen Asteraceae Species Native to Sudan and Bioactivity-Guided Isolation of Xanthanolides from *Xanthium Brasilicum*.“. Planta Med. 75 (12), 1363–1368. 10.1055/s-0029-1185676 19431098

[B62] Oboh G. Adebayo A. A. Ademosun A. O. Boligon A. A. (2017). In Vitro Inhibition of Phosphodiesterase-5 and Arginase Activities from Rat Penile Tissue by Two Nigerian Herbs (Hunteria Umbellata and Anogeissus Leiocarpus). J. Basic Clin. Physiol. Pharmacol. 28 (4), 393–401. 10.1515/jbcpp-2016-0143 28306529

[B63] Okba M. M. Sabry O. M. Matheeussen An Abdel-Sattar E. (2018). In Vitro Antiprotozoal Activity of Some Medicinal Plants against Sleeping Sickness, Chagas Disease and Leishmaniasis. Future Med. Chem. December. 10.4155/fmc-2018-0180 30511591

[B64] Okokon J. E. Nwafor P. A. (2009). Antiplasmodial Activity of Root Extract and Fractions of Croton Zambesicus. J. Ethnopharmacol. 121 (1), 74–78. 10.1016/j.jep.2008.09.034 18996464

[B65] Okpako L. C. Ajaiyeoba E. O. (2004). In Vitro and in Vivo Antimalarial Studies of Striga Hermonthica and Tapinanthus Sessilifolius Extracts. Afr. J. Med. Med. Sci. 33 (1), 73–75. 15490799

[B66] Osorio E. Arango G. J. Jiménez N. Alzate F. Ruiz G. Gutiérrez D. (2007). Antiprotozoal and Cytotoxic Activities in Vitro of Colombian Annonaceae. J. Ethnopharmacol. 111 (3), 630–635. 10.1016/j.jep.2007.01.015 17296281

[B67] Ouattara Y. Sanon S. TraorÃ© Y. Mahiou V. Azas N. Sawadogo L. (2006). Antimalarial Activity of *Swartzia madagascariensis* desv. (leguminosae), *Combretum glutinosum* guill. & perr. (combretaceae) and *Tinospora bakis* miers. (menispermaceae), Burkina Faso medicinal plants. Afr. J. Tradit. Complement. Altern. Medicines 3 (1), 75–81.

[B68] Pascolutti M. Campitelli M. Nguyen B. Pham N. Gorse A.-D. Quinn R. J. (2015). Capturing Nature’s Diversity. PloS One 10 (4), e0120942. 10.1371/journal.pone.0120942 25902039 PMC4406718

[B69] Peerzada A. M. Ali H. H. Naeem M. Latif M. Bukhari A. H. Tanveer A. (2015). Cyperus Rotundus L.: Traditional Uses, Phytochemistry, and Pharmacological Activities. J. Ethnopharmacol. 174, 540–560. 10.1016/j.jep.2015.08.012 26297840

[B70] Potterat O. Hamburger M. (2013). Concepts and Technologies for Tracking Bioactive Compounds in Natural Product Extracts: Generation of Libraries, and Hyphenation of Analytical Processes with Bioassays. Natural Prod. Rep. 30 (4), 546–564. 10.1039/c3np20094a 23459784

[B71] Potterat O. Hamburger M. (2014). Combined use of extract libraries and HPLC-based activity profiling for lead discovery: potential, challenges, and practical considerations. Planta Med. 80 (14), 1171–1181. 10.1055/s-0034-1382900 25098928

[B72] Räz B. Iten M. Grether-Bühler Y. Kaminsky R. Brun R. (1997). The Alamar Blue Assay to Determine Drug Sensitivity of African Trypanosomes (T.b. Rhodesiense and T.b. Gambiense) in Vitro. Acta Tropica 68 (2), 139–147. 10.1016/s0001-706x(97)00079-x 9386789

[B73] Rasmussen H. B. Christensen S. B. Kvist L. P. Karazmi A. (2000). A Simple and Efficient Separation of the Curcumins, the Antiprotozoal Constituents of Curcuma Longa. Planta Med. 66 (4), 396–398. 10.1055/s-2000-8533 10865470

[B74] Salehi B. Zakaria Z. A. Gyawali R. Ibrahim S. A. Rajkovic J. Shinwari Z. K. (2019). Piper Species: A Comprehensive Review on Their Phytochemistry, Biological Activities and Applications. Molecules 24 (7), 1364. 10.3390/molecules24071364 PMC6479398 30959974

[B75] Salem M. M. Davidorf F. H. Abdel-Rahman M. H. (2011). In Vitro Anti-Uveal Melanoma Activity of Phenolic Compounds from the Egyptian Medicinal Plant Acacia Nilotica. Fitoterapia 82 (8), 1279–1284. 10.1016/j.fitote.2011.08.020 21903153

[B76] Salih E. Y.A. Julkunen-Tiitto R. Lampi A.-M. Kanninen M. Luukkanen O. Sipi M. (2018). Terminalia Laxiflora and Terminalia Brownii Contain a Broad Spectrum of Antimycobacterial Compounds Including Ellagitannins, Ellagic Acid Derivatives, Triterpenes, Fatty Acids and Fatty Alcohols. J. Ethnopharmacol. 227, 82–96. 10.1016/j.jep.2018.04.030 29733942

[B77] Schmidt T. J. Khalid S. A. Romanha A. J. Ma Alves T. Biavatti M. W. Brun R. (2012). The Potential of Secondary Metabolites from Plants as Drugs or Leads against Protozoan Neglected Diseases - Part I. Curr. Med. Chem. 19 (14), 2128–2175. 10.2174/092986712800229023 22414103

[B78] Silva O. Gomes E. T. (2003). Guieranone A, a Naphthyl Butenone from the Leaves of Guiera Senegalensis with Antifungal Activity. J. Natural Prod. 66 (3), 447–449. 10.1021/np0204904 12662113

[B79] Simoes-Pires C. Hostettmann K. Haouala A. Cuendet M. Falquet J. Graz B. (2014). “Reverse Pharmacology for Developing an Anti-Malarial Phytomedicine. Example Argemone Mexicana Int. J. Parasitol.: Drugs Drug Resist. Includes Articles Two Meetings: Anthelmintics: Discovery Resist. 218–315 “Global Challenges New Drug Discovery Against Trop. Parasitic Dis. 316–357, 4 (3), 338–346. 10.1016/j.ijpddr.2014.07.001 PMC4266807 25516845

[B80] Straimer J. Gnädig N. F. Witkowski B. Amaratunga C. Duru V. Ramadani A. P. (2015). Drug Resistance. K13-Propeller Mutations Confer Artemisinin Resistance in Plasmodium Falciparum Clinical Isolates. Sci. (New York N.Y.) 347 (6220), 428–431. 10.1126/science.1260867 PMC4349400 25502314

[B81] Stuart K. Brun R. Croft S. Fairlamb A. Gürtler R. E. McKerrow J. (2008). Kinetoplastids: Related Protozoan Pathogens, Different Diseases. J. Clin. Invest. 118 (4), 1301–1310. 10.1172/JCI33945 18382742 PMC2276762

[B82] Suleiman M. H. A. (2015). “An Ethnobotanical Survey of Medicinal Plants Used by Communities of Northern Kordofan Region, Sudan. J. Ethnopharmacol. 176, 232–242. 10.1016/j.jep.2015.10.039 26519203

[B83] Tchoumbougnang F. Amvam Zollo P. H. Dagne E. Mekonnen Y. (2005). In Vivo Antimalarial Activity of Essential Oils from Cymbopogon citratus and Ocimum gratissimum on Mice Infected with Plasmodium berghei. Planta Med. 71 (1), 20–23. 10.1055/s-2005-837745 15678368

[B84] Tona L. Cimanga R. K. Mesia K. Musuamba C. T. De Bruyne T. Apers S. (2004). In Vitro Antiplasmodial Activity of Extracts and Fractions from Seven Medicinal Plants Used in the Democratic Republic of Congo. J. Ethnopharmacol. 93 (1), 27–32. 10.1016/j.jep.2004.02.022 15182900

[B85] Traore M. Diane S. Diallo M. Balde E. Balde M. Camara Aïssata (2014). In Vitro Antiprotozoal and Cytotoxic Activity of Ethnopharmacologically Selected Guinean Plants. Planta Med. 80 (15), 1340–1344. 10.1055/s-0034-1383047 25180493

[B86] Varpe S. S. Juvekar A. R. Bidikar M. P. Juvekar P. R. (2012). Evaluation of Anti-Inflammatory Activity of Typha Angustifolia Pollen Grains Extracts in Experimental Animals. Indian J. Pharmacol. 44 (6), 788–791. 10.4103/0253-7613.103303 23248413 PMC3523511

[B87] Waako P. J. Gumede B. Smith P. Folb P. I. (2005). The in Vitro and in Vivo Antimalarial Activity of Cardiospermum Halicacabum L. and Momordica Foetida Schumch. Et Thonn. J. Ethnopharmacol. 99 (1), 137–143. 10.1016/j.jep.2005.02.017 15848033

[B88] Waterman P. G. Khalid S. A. (1980). The Major Flavonoids of the Seed of Tephrosia Apollinea. Phytochemistry 19 (5), 909–915. 10.1016/0031-9422(80)85137-5

[B89] World Health Organisation (2018). World Malaria Report. Geneva: WHO.

[B90] WHO | Epidemiology (n.d). WHO. Accessed October 22, 2019 http://www.who.int/chagas/epidemiology/en/.

[B91] WHO | World Health Organization (n.d). WHO. Accessed December 4, 2019 http://www.who.int/neglected_diseases/diseases/en/.

[B92] WHO Expert Committee on Malaria Twentieth Report (n.d). Accessed April 28, 2019 https://apps.who.int/iris/handle/10665/42247.

[B93] Wiser M. F. Eaton J. W. Sheppard J. R. (1983). A Plasmodium Protein Kinase That Is Developmentally Regulated, Stimulated by Spermine, and Inhibited by Quercetin. J. Cell. Biochem. 21 (4), 305–314. 10.1002/jcb.240210407 6654993

[B94] Witschel M. Rottmann M. Kaiser M. Brun R. (2012). Agrochemicals against Malaria, Sleeping Sickness, Leishmaniasis and Chagas Disease. PloS Neglected Trop. Dis. 6 (10), e1805. 10.1371/journal.pntd.0001805 PMC3493374 23145187

[B95] Wulf J. S. Rühmann S. Rego I. Puhl I. Treutter D. Zude M. (2008). Nondestructive Application of Laser-Induced Fluorescence Spectroscopy for Quantitative Analyses of Phenolic Compounds in Strawberry Fruits (Fragaria x Ananassa). J. Agric. Food Chem. 56 (9), 2875–2882. 10.1021/jf072495i 18416555

[B96] Yagi S. Babiker R. Tzanova T. Schohn H. (2016). Chemical Composition, Antiproliferative, Antioxidant and Antibacterial Activities of Essential Oils from Aromatic Plants Growing in Sudan. Asian Pacific J. Trop. Med. 9 (8), 763–770. 10.1016/j.apjtm.2016.06.009 27569885

[B97] Zininga T. Ramatsui L. Makhado P. B. Makumire S. Achilinou I. Hoppe H. (2017). (-)-Epigallocatechin-3-Gallate Inhibits the Chaperone Activity of Plasmodium Falciparum Hsp70 Chaperones and Abrogates Their Association with Functional Partners. Mol. (Basel Switzerland) 22 (12), 2139. 10.3390/molecules22122139 PMC6149709 29206141

[B98] Zirihi GuédéNoël Mambu L. Guédé-Guina Frédéric Bodo B. Grellier P. (2005). In Vitro Antiplasmodial Activity and Cytotoxicity of 33 West African Plants Used for Treatment of Malaria. J. Ethnopharmacol. 98 (3), 281–285. 10.1016/j.jep.2005.01.004 15814260

[B99] Zulfiqar B. Jones A. J. Sykes M. L. Shelper T. B. Davis R. A. Avery V. M. (2017). Screening a Natural Product-Based Library against Kinetoplastid Parasites. Mol. (Basel Switzerland) 22 (10), 1715. 10.3390/molecules22101715 PMC6151456 29023425

